# How Epigenetics Can Enhance Pig Welfare?

**DOI:** 10.3390/ani12010032

**Published:** 2021-12-24

**Authors:** Arthur Nery da Silva, Michelle Silva Araujo, Fábio Pértille, Adroaldo José Zanella

**Affiliations:** 1 Department of Preventive Veterinary Medicine and Animal Health, School of Veterinary Medicine and Animal Science, Campus “Fernando Costa”, University of São Paulo, Pirassununga 13635-900, Brazil; arthur_nery@usp.br (A.N.d.S.); msa.vet@usp.br (M.S.A.); 2 Department of Biomedical & Clinical Sciences (BKV), Linköping University, SE-58183 Linkoping, Sweden

**Keywords:** swine, stress, biomarkers, DNA methylation, epigenetics, welfare certification

## Abstract

**Simple Summary:**

In the pig industry, new market trends and consumer demands have emerged over the past decades, which includes increased concerns about how animals are raised on farms. As a consequence of consumers’ concerns, technologies capable of predicting animal welfare on farms have been explored. One of the technologies that are permeating the frontier of knowledge in this area are epigenetic biomarkers. Epigenetic biomarkers are biochemical markers surrounding the genome, which may be able to predict the exposures that individuals had during their lifetime. These markers represent an advance in the molecular level accuracy to support the current welfare indicators. In this literature review focused on pigs, we show some studies already carried out, we performed an integrative analysis of the already reported genes surrounding epi-biomarkers, and we highlight the benefits of investing efforts in this research field to enhance animal welfare and consumers’ trust.

**Abstract:**

Epigenetics works as an interface between the individual and its environment to provide phenotypic plasticity to increase individual adaptation capabilities. Recently, a wide variety of epi-genetic findings have indicated evidence for its application in the development of putative epi-biomarkers of stress in farm animals. The purpose of this study was to evaluate previously reported stress epi-biomarkers in swine and encourage researchers to investigate potential paths for the development of a robust molecular tool for animal welfare certification. In this literature review, we report on the scientific concerns in the swine production chain, the management carried out on the farms, and the potential implications of these practices for the animals’ welfare and their epigenome. To assess reported epi-biomarkers, we identified, from previous studies, potentially stress-related genes surrounding epi-biomarkers. With those genes, we carried out a functional enrichment analysis of differentially methylated regions (DMRs) of the DNA of swine subjected to different stress-related conditions (e.g., heat stress, intrauterine insult, and sanitary challenges). We identified potential epi-biomarkers for target analysis, which could be added to the current guidelines and certification schemes to guarantee and certify animal welfare on farms. We believe that this technology may have the power to increase consumers’ trust in animal welfare.

## 1. Introduction

Animal welfare has become a public concern globally [1,2,3]. This spans companion animals, sporting events, laboratory experimentation and livestock industries. There is a growing demand for products that meet high animal welfare standards, which the industry is strongly committed to meeting [4]. Consequently, the biggest worldwide pork exporters have adjusted their production chain to align with animal welfare demands [5]. For instance, in the UK and in the European Union, gestation crates have been banned [6], and a similar trend has been observed in Brazil with the recent directive to establish good animal management and welfare practices for commercially-raised pigs [7].

An important topic that has been attracting the attention of the animal protein industry is the animal welfare certification, which is demanded by importers. This stems from the fact that consumers want to be aware of their purchased meat’s origin, and the animal welfare conditions within the production systems in which these animals are reared [8]. In this regard, biotechnological approaches have been applied to ensure with great accuracy that this demand is met. Companies that go to farms to check behavior, management, biosecurity, and animal welfare play a fundamental role in accomplishing the current goals of ensuring adequate animal welfare within the industry. Moreover, these companies provide certification for pig farms that comply with all animal welfare guidelines. However, once the technician/auditor has left the farm, it is difficult to guarantee that ap-propriate procedures will be constantly applied. Even though practices like improper handling may cause variations in the organoleptic characteristics (such as color, brightness, odor, texture, and taste) and/or chemical composition (such as pH, water holding capacity or color) of the meat [9,10], these changes are seldom noticed by the consumer. Moreover, it directly infers on the moral values of the final consumer who is purchasing the product certified for animal welfare, because he/she might pay for a product that does not meet his/her expectations of good welfare. Thus, the establishment of the animal’s physiological and molecular information, which ensures consumers that the animals have been bred and raised in compliance with a set of pre-established welfare standards, not only benefits customers by providing the tools to make informed choices about their purchases, but also allows the market to aggregate more value to their products. This information could be certified by a stamp that translates physiological and molecular parameters of the animals into a “handling score”, for example.

One area of study that links the effects of the environment and intrinsic factors on individuals is epigenetics [11]. Epigenetic studies have been used in many fields of research, such as pharmacology [12], nutrition [13], and welfare [14] across species. Among the numerous fields that epigenetics permeates, efforts to identify epigenetic markers of long-term stress in production animals is currently a hot topic within the animal welfare field [14,15,16,17].

In this article, we provide an overview of some of the investigations already carried out, as well as challenges and potentials associated with this approach. This compilation of peer-review articles explored epigenetic markers in animal welfare research. Our aim is to encourage researchers to extensively investigate potential new paths for the development of a robust molecular tool for animal welfare certification. This tool, together with a careful human inspection, may have the power to greatly increase the precision of current welfare indicators and boosts the credibility of pig producers that comply with welfare guidelines, as well as empowers meat consumers concerned about animal welfare and food quality. For this purpose, we have organized this literature review in such a way that we present the current consumers’ demands on animal protein, the relevance of housing systems for ensuring animal welfare, the methods that are currently used to measure pig welfare, what are the outcomes of poor animal welfare for the epigenome of pigs, and how it can be used as an epigenetic biomarker of stress.

## 2. Livestock Demands

Livestock production is expected to continue to increase to meet growing demand for animal products [18]. However, this is expected to result in poorer animal welfare [19]. Recently, more and more consumers have raised concerns about the systems and conditions in which their food is produced, potentially driving new trends focused on ethical production [20]. Furthermore, in 2015, the United Nations implicitly set animal welfare as a point of synergy for the sustainable development of food production at the global level [21,22].

Farming activities are no longer seen simply as for the production of food [23]. Farming is increasingly viewed through the lens of “one welfare”, where farmers influence the health of the environment, of the farm animals under their care, and consumers who buy their products [24]. This new demand came from consumers who dictate what kinds of products they want to eat based on their concepts of quality and safety [9]. In addition, these reflections on “one welfare” generated the possibility of increasing monetization for the farmer [25], because some certified products are more expensive for the final consumer. 

Considering these demands and market opportunities, the management and housing systems of the animals play a fundamental role to guarantee animal welfare. To illustrate the importance of housing systems, we present in the next section some of the implications observed in the field that are of relevance to pig welfare.

## 3. Housing Systems

Meeting minimum necessary housing requirements may not be a major challenge in extensive production and for small pig farmers who normally target their product to the local market or for self-consumption. However, in intensive systems, even the minimum requirements can be challenging as they must follow international rules to meet all the animals’ needs and market expectations, which includes animal welfare [26,27]. In this regard, one of the most important factors to provide adequate welfare is the housing system.

Currently, there are different setups of conventional housing systems for pigs, which include crates, indoor group housing, and outdoor systems—each of which carries ad-vantages and disadvantages for pigs and farmers. For example, an indoor group housing system is commonly characterized as posing physical challenges to veterinary assistance and to animal feeding, in comparison to crates [28,29]. However, in terms of behavior indicators, indoor group housing tends to result in better welfare [28,30,31]. Another relevant issue involved in the livestock industry is the management of organic and pharmaceutical residues [32], sustainable use of the land [33], and financial costs, which also vary by housing system.

Despite the variety of housing system possibilities for pigs, each with its respective pros and cons, the welfare conditions currently found in some systems are considered precarious and requiring immediate change [34]. For example, the welfare of crated sows in several countries is deemed very poor. It was reported that sows kept in crates have limited expression of natural behavior, which leads to neurological dysfunctions and lameness [34,35,36,37,38]. Alternatively, group housing allows animals to express social behavior, which is associated with decreased agonistic interactions, reduced stereotypies, and improved cognition [35]. However, a frequent concern reported by pig farmers is the innate social aspect of hierarchy, which can be a challenge when housing sows in groups, as hostile behavior may arise from social disputes and result in compromised welfare and production outcomes [39].

In this scenario, even though indoor group housing and outdoor systems may represent challenges of their own and the transition may be difficult or costly for farmers, pressures by legislators [6,7] and demands by consumers are decisive [40,41]. Therefore, in the next topics of our review we point out potentially useful approaches to certify animal welfare.

## 4. Animal Welfare Indicators

Broom [42] suggested two ways to access behavioral indicators of poor animal welfare. The first focuses on individual failure to cope with the environment. This is an easily identifiable indicator by the pig farmers, since it aligns with increases in mortality and productivity declines. An example of this situation is when the environment in which the individual is raised is poor and leads animals to develop abnormal behaviors or diseases. Therefore, it is impossible for the animal to express its full potential and, consequently, significant economic losses are inevitable. The second type of indicator focuses on how individuals cope with environmental adversity. These indicators are usually more difficult for farmers to assess because they involve physiological outcomes in the animals, such as cardiac, respiratory, gastrointestinal, and hormonal changes. In general, these indicators do not lead to death, but they may reduce the welfare and performance of the animals [42].

To use quantitative measures to assess the level of animal welfare, biochemical markers have been employed at the experimental level. However, these approaches remain insufficient, because they can only identify the animal’s biochemical profile in a limited time frame, compromising its applicability in the industry [43]. For example, these indicators may reflect the poor welfare experienced within just a few hours or days before the measurement, depending on its half-life, and is not a reliable account of the animal’s life trajectory, like the effects of weaning or gestating in crates. Moreover, these markers are usually limited to specific tissues or fluids, which limits their applicability to a broad suite of welfare problems. For example, creatine kinase (CK) is an enzyme involved in the citric acid cycle, producing energy in the mitochondria. Many studies have shown its consistency as a biomarker in the tissue of farm animals raised under different levels of welfare [44,45,46,47]. These studies suggested that animals with high CK at the time of slaughter were previously subjected to stressful situations [44,45,46,47]. Likewise, the measurement of serum lactate concentrations has also shown to be a promising biomarker, mainly for measuring pre-slaughter stress, despite its short half-life [45,48].

Hormones are another known indicator of animal welfare [49]. Cortisol, for example, is a glucocorticoid hormone released under stressful situations, capable of affecting sever-al physiological pathways, including the hypothalamic–pituitary–gonadal axis [50,51]. This hormone has a recognized importance in the evolution and physiological adjustment of many species [52]. However, cortisol also plays a fundamental role as a biochemical marker of acute or medium-term stress in animal production [49]. Currently, different research groups have been striving to develop consistent endocrine profiles for chronically stressed animals and its outcomes in the organism [53]. However, cortisol measurement techniques remain insufficient for the purpose of welfare assessment [53,54]. Sampling techniques usually provide cortisol concentrations only from a few days or weeks [49,55], which limits its practical use. Lastly, cortisol level variations are not fully understood as a stress indicator because both positive and negative exposures can affect its fluctuation [56]. Furthermore, its use as an indicator of stress has been questioned [54,56].

Studies have shown that chronic stress alters the expression of key enzymes involved in stress susceptibility [57,58] and spine plasticity [59], which can cause specific epigenetic marks in the genome and behavioral changes [57,58,59,60]. These triggered modifications around the genome do not produce genetic changes, however, they can alter gene expression and protein transcription. An attempt to predict the proteomic profile of animals raised under good or poor welfare conditions was reviewed by Mouzo et al. [43]. They highlighted the advantages of using proteomic approaches to predict animal welfare according to the animal protein biochemical composition. For example, the study made important considerations about the protein profile of pale soft exudative (PSE) meat, which is one of the main depreciation factors of pork meat, and how proteomic approaches can be used to predict it. However, proteomic approaches have provided a landscape view of the gene expression and its consistency is quite variable because there is a wide variety of mechanisms describing post-translational mechanisms shaping the protein production [43]. Consequently, challenges can arise for the long-term stress assessment using proteomic approaches [61]. By comparing epigenetics and proteomics, proteomics was recently shown to be a preferable approach to assess short-term stress, whereas epigenetics might be a better forecaster for early prediction of stress susceptibility and a suited approach to be added into the animal breeding schemes [61]. Taking this into consideration, we hypothesize that the investigation of epigenetic mechanisms may offer a valuable attempt to identify signatures above the genome of animals raised under different welfare conditions, which may have a greater power to predict its life-long welfare.

## 5. Epigenetics

The term “epigenetics” was first coined by Waddington in 1942, who suggested the interaction of external factors with the genome as “epigenotype”, and that this interaction could affect the development of the individuals [62]. Recently, epigenetics was recognized as an interface in the transition from the genetic code into a functional mRNA that may be traduced into a protein [11]. Epigenetics consists of a heritable pattern of chemical alterations on DNA that can modulate gene expression without alterations in the base pairs structure of the DNA double strand [63]. In addition, these specific patterns are maintained and inherited during the mitotic, and possibly meiotic, divisions of the cells [63].

The importance of epigenetics is not limited to a cellular perspective only. Epigenetics has a huge and decisive role in evolution and speciation [64,65,66]. Moreover, epigenetic mechanisms can affect the gene expression of one or more generations and influence the adaptation of individuals to a specific environment [62]. Therefore, Hyde et al. [11] suggested that epigenetics works as the interface between the individual and its environment to provide phenotypic plasticity to increase their adaptation capabilities [11]. Some of the epigenetic mechanisms that act in modulating gene expression are DNA methylation [15], DNA acetylation [67], histone modifications [68], nucleosome repositioning [69], and small interfering RNAs [16]. These biological mechanisms help cells to differentiate not only morphologically, but also functionally, controlling the genomic regions that will be accessed and/or expressed. DNA methylation is the most investigated mechanism in epigenetics [11]. This mechanism involves an addition of 5′ methyl to the cytosine followed by guanine (CpG) in the DNA chain, and this reaction is generally associated with repression in gene expression [11]. However, several studies reported an enormous dependence on the methylation location regarding the gene to predict their potential effect on the gene expression and/or regulation if, for example, the methyl marks are in a promoter, intronic or coding region of the genome. In addition, the level of methylation of one CpG is also important, because it can also infer modulation of the gene expression [70]. Then, to examine this amount of information, bioinformatics analysis using differential methylation regions (DMR) is used to compare hypo or hypermethylated patterns among individuals [71].

The interaction among animals and their environments was an issue of discussion even before the theory of evolution through natural selection [72]. However, the role of genetic factors at the molecular level and their environmental interactions are still premature [38,73,74,75,76,77]. When the individual is exposed to an environmental experience, it is possible to determine what the epigenetic effects generated are [78], and methylation is a promising biomarker to detect and evaluate these effects [17]. In the last decades, valuable efforts have been made to understand the epigenetic differences in the genome of experimental animals, which is the topic of our next session of this review.

## 6. Epigenetic Assessment in Mammals

Weaver et al. [79] provided the first evidence that maternal care could produce persistent changes in epigenetic patterns in rats, which included DNA methylation and chromatin remodeling analyses. They revealed a mechanism for the long-term effects of maternal care in the progeny, caused by a stressful challenge during early life. The authors showed that the descendants who received more maternal care demonstrated low reactions to stress in adulthood, while offspring that received less maternal care were more susceptible to stress. This happened because the epigenomes of the adult rats exhibited different patterns, specifically the glucocorticoid receptor gene promoter in the hippocampus, which possibly affected the hypothalamic–pituitary–adrenal axis and its responses to stressful situations [79]. In addition, Champagne [80] elucidated the evidence for the generational transmission of maternal care and mechanisms underlying transmission [80].

**Figure 1:**
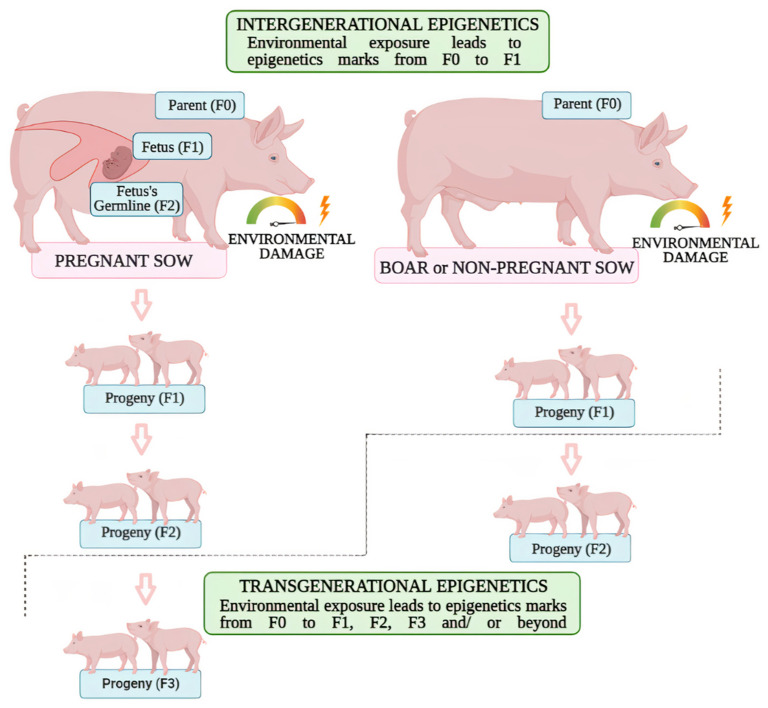
Intergenerational and transgenerational epigenetic inheritance in porcine models. When a swine (F0) is exposed to an environmental insult, somatic and germ cells will potentially affect their epigenome. In addition, if it is a pregnant sow, the fetus (F1) and its germ cells—which will give rise to a next generation—will be directly affected (F2). So, if these epigenetic marks contained in the fetus’s germ cells remains for subsequent generations (F3 and beyond), there will be a transgenerational epigenetic event.

Intergenerational and transgenerational epigenetics is one of the most discussed topics in the field of epigenetics inheritance. Current literature supports the understanding that the transmission of epigenetics marks from one generation (F0) to the next (F1) represents an intergenerational event; while the transmission acquired in F0 that is transmitted to the third or fourth generation (F2 for males or F3 for females) can be considered as transgenerational epigenetic event [81]. In other words, different numbers of generations are directly affected by environmental insults in males and non-pregnant females when compared to pregnant females. This happens because the majority of female mammals have their oocytes produced during their fetal development [82,83]. For example, the por-cine female fetuses’ gonads differentiate into an ovary containing gamete cells by day 30 of pregnancy and by mid-gestation primordial follicles are already recognizable [84]. Consequently, if a pregnant female suffers a potential epigenotoxic environmental exposure, not only will the fetus be directly affected by this environmental insult, but also the germ cells of the fetus. In summary, three generations can be epigenetically affected by an environmental insult: F0 (somatic and germ cells of the pregnant female), F1 (fetus), F2 (germ cells of the fetus) [81,85,86]. Figure 1 was provided for exemplification, using the porcine model.

Although the most characterized epigenetic studies have been conducted in small experimental animals, such as rodents [79,87,88,89], insects [13], and worms [90], a representative number of studies have been done on domestic animals, such as pigs [89,91,92,93,94,95]. In the following section, we will discuss in detail studies focused on pigs as models in epigenetic investigations and draw attention to the role of stress as an epigenetic modulator.

## 7. Epigenetics Studies in the Porcine Model

An important role of epigenetics in gene expression of productive [15,16,96,97,98] and reproductive traits of pigs [99,100,101] has been demonstrated. Moreover, recent work has demonstrated the impact of several factors on the epigenome of the domestic pig such as the influence of nutrition on the pregnant sow epigenome [93,94,102], the impact of exposure of pregnant sows to chemicals on its offspring’s epigenome [89,91], and the influence of the year´s season on the epigenome of the boars’ ejaculate and its fertility [95,103,104,105].

Studies have indicated that the management of pigs during gestation promotes changes in their offspring’s behavior [38,73,75,106]. However, to the best of our knowledge, stressful events in pig farming, such as the effect of gestation crates, lameness, and social isolation have not been explored by epigenetic studies in pigs [107,108]. These studies would be valuable for both animal welfare and the industry. Likewise, these epigenetic investigations would also be valuable for translational studies, as the porcine model is a well-known standard for human studies [109,110]. 

**Figure 2:**
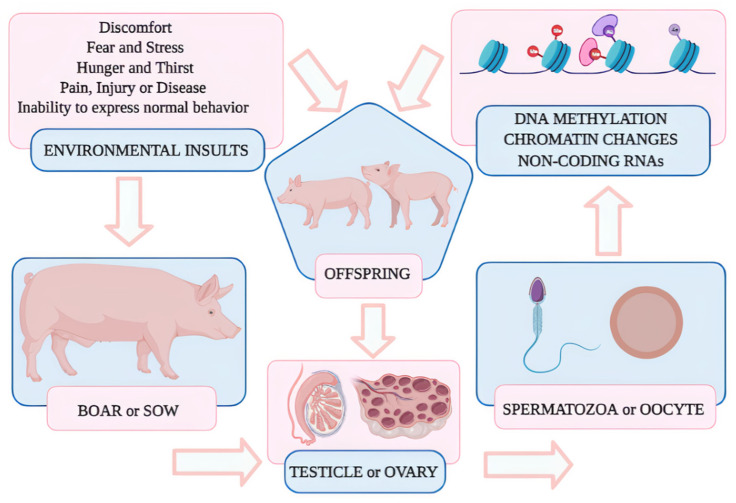
A possible pathway of epigenetic transmission in industrial pig production systems.

A possible mechanism by which epigenetics may act is shown in Figure 2. We hypothesize that negative situations where pigs are exposed to at challenging production systems can affect somatic and germ cells. Thereby, epigenetic patterns could be transmit-ted from parents to their offspring, but for that to happen, it needs to be present somehow in their germline strain (sperm or oocytes) epigenome. Therefore, not only does the individual accumulate epigenetic marks during its life, but it also inherits different patterns from its parents.

Valuable efforts have been made using the porcine model in epigenetic studies (Table 1). However, to the best of our knowledge, there are few epigenetic studies related to the daily challenges of pig farming, such as inadequate housing, painful procedures, heat stress, or other stressful situations. First, Collier et al. [111] highlighted the role of maternal stress and its potential for the inheritance of epigenetic changes by future generations [111]. Nevertheless, they did not use an epigenetic approach to support their suggested mechanism. Their study focused on measuring cortisol, interleukins, cytokines, and other physiological biomarkers. Then, Schachtschneider et al. [112] demonstrated that early-life challenges, such as iron deficiency and porcine reproductive and respiratory syndrome virus (PRRS), were reported to alter DNA methylation and expression of key genes related to hippocampal plasticity, which can cause long-term cognitive damage [112]. Recently, Kasper et al. [61] published a literature review showing the potential of omics approaches in the development of biomarkers in pig production, including epigenetics, and how it could positively impact the issue of tail biting.

The absence of disease is a welfare demand, and epigenetic studies have also revealed interesting contributions to explain pathophysiological mechanisms of infections by microorganisms. A study performed by Sajjanar et al. [117] showed the role of the DMRs in regulating genomic regions of porcine mammary epithelial cells infected by *Escherichia coli* strains when compared with non-infected cells. They identified significant DMRs, hypomethylated in the cells infected with *E. coli*, in the promoter region of the *SDF4*, *SRXN1*, *CSF1* and *CXCL14* genes. Using functional network analysis, the authors also reported that these genes are related to innate and adaptative immune response pathways [117]. In addition, a study carried out by Simões et al. [118] clarified how an African swine fever infection can impair the subnuclear domains and chromatin architecture of infected cells, compromising gene expression, and favoring viral dissemination. This mechanism is part of an emergent field of studies, which have shown that some viruses subvert cellular epigenetic mechanisms and recruit host transcription factors to their benefit by changing chromatin structure [118,119]. 

Using muscle tissue samples from a heat stress-exposed group and an unexposed group of pigs, the study performed by Hao et al. [15] showed that the methylation level of the heat-stressed group was significantly lower than what was found among the control group for some genomic regions. Moreover, they showed that the DMRs were located around important genes related to cell development, which may play a role in muscle performance and function. In another study, Hao et al. [16] showed different microRNA expression profiles in pigs affected by chronic heat stress, which probably affected gene expression at a post-transcriptional level. Lastly, the study conducted by Ponsuksili et al. [97] showed differences in the epigenetic patterns of muscle cells from different pig breeds and their role in the development of the muscle phenotype.

Table 1 summarizes some important findings and advances using pigs as an experimental model in epigenetic related studies. We selected only studies originally published in peer-reviewed journals that addressed relevant information on epigenetics over the past 10 years. To classify the studies according to epigenetic mechanisms in the table, we used the concepts recommended by Lacal and Ventura [81], Tuscher and Day [86], and John and Rougeulle [120] as a basis. They suggested that only changes in the F3 generation in females, and F2 in males [81,86], can be defined as a “transgenerational epigenetic inheritance” event. In addition, the concept of “intergenerational epigenetics” was used to define studies that addressed exposures that led to epigenetic changes in the somatic tissues of the F1 offspring but did not persist/or was not tested in the F2 or F3 generations [81,86]. Finally, the term “direct exposure” was used to define studies that investigated epigenetic marks in a single generation (F0) [120].

## 8. Gene Network Analysis on Stress in Pigs

Although there is substantial evidence regarding the suitability of methylation as a molecular marker to predict pig welfare [61], the findings are still premature and further research will be needed. To the best of our knowledge, the number of articles reporting DMRs in the context of compromised welfare is limited [15,96,102,112,117]. In addition, the results are quite variable in terms of specific affected genes. However, this is expected, since the experimental contexts are different, and include intrinsic and extrinsic variations of individuals, such as genotypic variability and tested stress models, for example. In addition, the laboratorial and statistical approaches performed for DMR identification across the experiments are also variable among the previous published studies.

**Figure 3:**
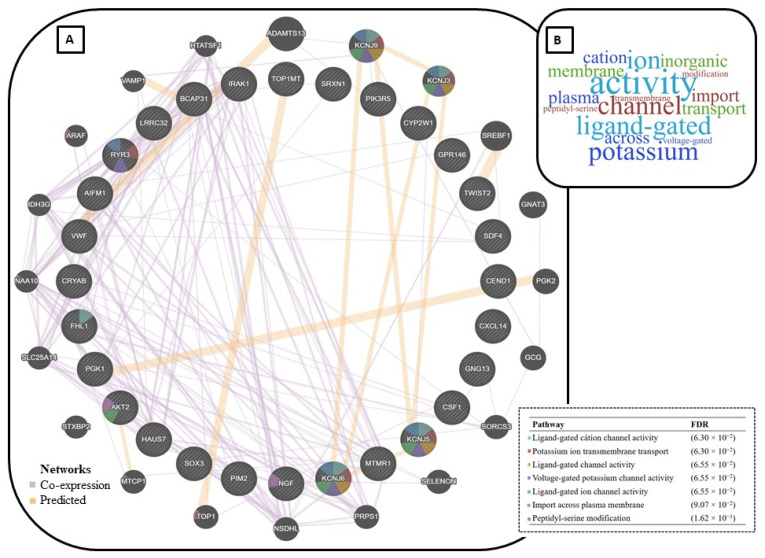
Prediction of the gene network (**A**) and most cited pathways (**B**) in which the genes play a role using the human genome as a reference.

Therefore, to explore the common biological functions performed by the previously identified stress-related genes, we performed an integrative analysis considering these previously identified epi-markers. A functional enrichment analysis was carried out with the 28 DMR-related genes identified as significant from each one of the previous studies when subjected to different stress conditions (Table 2). This analysis was performed by gene enrichment in the category of co-expression and predicted network using GeneMania web environment [121] (acessed on 14 September 2021). The integration of the genes and its most cited molecular functions can be seen in Figure 3. We summarized the main identified functions of the genes using a word cloud web-tool (https://www.wordclouds.com/ accessed on 7 October 2021), which takes into consideration the number of times a word is identified to output it with its proportional font size.

We found 28 candidate genes in the literature that are connected in our stress-related epi-gene network. Moreover, another 20 genes emerged as connected to this network and consequently are potential candidate genes for this same trait. For example, the genes *KCNJ3* and *KCNJ9* were already reported to be associated with neurologic dysfunctions, such as schizophrenia [122] and hemiplegia [123], respectively, in humans. Interestingly, the genes enriched 7 pathways related to transport mechanisms across the cell membrane. Moreover, the genes *KCNJ6*, *KCNJ5*, *FHL1*, *AKT2*, *NGF* and *RYR3* from the main core of the network were enriched for the regulation of potassium channel activity pathway. Those genes were previously reported to be relevant when analyzing heat stress [15] and animal exposition to sanitary challenges [112], which are two of the hot topics in animal welfare in pig research [15,112]. From the gene network (Figure 3A), most of the pathways (Figure 3B) identified (FDR ≤ 0.19) by the enriched genes play a role in the regulation of transmembrane transport and basic cell signaling processes. Notably, the regulation of potassium channel activity was the top pathway in our analysis. Potassium ion transmembrane transport activity is one of the most important mechanisms for cell physiology in a wide variety of tissues [124]. Consequently, their malfunction has been associated with a wide variety of pathologies [125,126]. Dysfunction of potassium channels in the nervous system has been associated with health issues like epilepsy, episodic ataxia, Alzheimer’s disease, Parkinson’s disease, and schizophrenia [125]. This happens because these channels play a central role in cell excitability [127], hormone secretion [128], cell proliferation [129], and apoptosis [130]. To the best of our knowledge, studies have not identified a clear relationship between up or downregulation of potassium ion transport activity and brain neurologic dysfunction in pigs, but in the mice model, pieces of evidence have been raised [131,132]. After inducing a group of mice to acute stress and assessing their behavior response to this situation, Guo et al. [132] identified that acute stress-induced a significant reduction in calcium-potassium channels in the amygdala of the stressed mice. This molecular pathway and this source of tissue may be a potential candidate to assess long-term information of pigs, and mammals in general, exposed to stressful situations.

Lastly, epigenetic studies have enormous potential to provide life-long information associated with the animal’s attempts to cope with its environment, which is central to the most cited animal welfare definition [42], when the mechanisms for the domestic pig and other species will be better understood.

## 9. Conclusions

In the last decade, the interest in the epigenetic field has exponentially increased and has shown enormous potential in answering scientific questions in different areas. In the animal welfare field applied to livestock animals, valuable attempts have been made to identify putative epigenetic biomarkers of stress. Furthermore, this knowledge may have the potential to increase productivity, health, and meat quality. Thus, considering the latest evidence, using epigenetics as a tool to certify animal welfare may be one of the new trends in the pig industry. In this study, we brought together the latest findings in the area of epigenetic markers in studies of well-being in pigs. In addition, we have provided a list of potential genes for target analysis, which could be used in methylated DNA sequencing studies using Bisulfite conversion, which can enhance the application of this technology in animal breeding schemes. We aim for a future in which epigenetics will be included as a measure for physiological and animal management parameters, which will help to improve our decision-making into how to improve housing systems, food quality, and the production system of pigs in general. At this moment, findings remain quite premature for assuring the development of an epigenetic panel of biomarkers capable of predicting life-long welfare in commercially raised pigs. However, future studies will not only elucidate mechanisms in the stress response but are also likely to increase the number of publications to form a panel of biomarkers capable of predicting animal welfare.

## Figures and Tables

**Table 1:** Studies related to swine epigenetics in different experimental contexts.

Mechanism	Generation	Swine Model	Context	Reference
Direct exposure	F0	Boars	Investigation of methylation patterns of testis samples and their relationship with the boar taint flavour.	[104]
		Boar’s semen	Correlation between different parameters of sperm DNA integrity and their methylation patterns.	[103]
			DMRs are more efficient at discerning the fertility of boars’ ejaculate than single nucleotide polymorphisms (SNPs) using reduced representation of the methylated DNA.	[105]
		Gilts	The epigenetic dynamic in hypothalamus-pituitary-ovary axis and its tissue-specific manner to establish the biological functions.	[113]
			The dynamics of hypothalamic methylation at puberty.	[114]
			Long-term effects of endocrine-active compounds on corpus luteum of swine females exposed during early life period.	[115]
		Porcine embryos	Investigation of the effects of histone deacetylase inhibitors on the in vitro development of porcine embryos derived from somatic cell nuclear transfer.	[100]
		Porcine oocytes	The effects of vitamin C in the regulation of global epigenetic modifications at DNA, RNA and histones levels and its potential for oocyte maturation and developmental competence.	[99]
		Porcine ovary	Epigenetic mechanisms of ovarian development during the transition from puberty and sexual maturation.	[116]
Intergenerational epigenetics	F0–F1	Pregnant sows and its offspring	Effects of exposure to low or high doses of estrogen during pregnancy and its role in female reproductive organs.	[89]
			The immediate and long-term effects of maternal dietary protein affecting gene expression of offspring.	[93]
			Restriction and excess dietary protein during pregnancy alters the offspring’s epigenetic marks and influences gene expression.	[94]
		Boar’s semen and sow’s placenta	The role of breeding season in altering epigenetic components of the placenta and its consequences to foetal development.	[101]
Transgenerational epigenetics	F0–F2	Boars	Transgenerational response of a methyl-enriched diet to boars and its responses on carcass traits, gene expression and DNA methylation.	[95]

**Table 2:** Effect of stress on pigs’ genome subjected to different environmental insults. The approaches used to access the methylated DNA was whole genome bisulfite sequencing (WGBS) or reduced representation bisulfite sequencing (RRBS).

Stress Source	Analysed Sample	Approach	Effect of Stress	Biomarker	Reference
Heat stress	*Longissimus dorsi* muscle	WGBS	DMRs in important genes involved in muscle development, metabolism, immunity, and stress response.	*RYR3*, *PGK1*, *CRYAB* and *FHL1C*	[15]
Intrauterine insult	Small intestine	RRBS	DMRs in several genes involved in cell development and immunity.	*IRAK1*, *AIFM1*, *PIM2*, *BCAP31*, *MTMR1*, *SOX3*, *TWIST2* and *HAUS7*	[96]
Sanitary challenge	Mammary epithelial cell	RRBS	DMRs in functional genes of the innate and adaptive immune response.	*SDF4*, *SRXN1*, *CSF1* and *CXCL14*	[117]
	Mid intestine	RRBS	DMRS in genes involved in structural pathways of the cells with outcomes in the immature prenatal intestine.	*CYP2W1*, *GPR146*, *TOP1MT* and *CEND1*	[102]
	Hippocampus	RRBS	DMRs in genes associated with blood brain barrier permeability and regulatory T-cell activation, which are reported to cause reductions in cognitive development.	*VWF*, *LRRC32*, *NGF*, *GNG13*, *PIK3R5*, *KCNJ6*, *KCNJ5* and *AKT2*	[112]

## Data Availability

Not applicable.
